# Long-term impact of systemic bacterial infection on the cerebral vasculature and microglia

**DOI:** 10.1186/1742-2094-9-146

**Published:** 2012-06-27

**Authors:** Ursula Püntener, Steven G Booth, V Hugh Perry, Jessica L Teeling

**Affiliations:** 1 Centre for Biological Sciences, University of Southampton, South Lab and Path Block, MP840, Southampton General Hospital, Tremona Road, Southampton, SO16 6YD, UK

## Abstract

**Background:**

Systemic infection leads to generation of inflammatory mediators that result in metabolic and behavioural changes. Repeated or chronic systemic inflammation leads to a state of innate immune tolerance: a protective mechanism against overactivity of the immune system. In this study, we investigated the immune adaptation of microglia and brain vascular endothelial cells in response to systemic inflammation or bacterial infection.

**Methods:**

Mice were given repeated doses of lipopolysaccharide (LPS) or a single injection of live *Salmonella typhimurium*. Inflammatory cytokines were measured in serum, spleen and brain, and microglial phenotype studied by immunohistochemistry. To assess priming of the innate immune response in the brain, mice were infected with *Salmonella typhimurium* and subsequently challenged with a focal unilateral intracerebral injection of LPS.

**Results:**

Repeated systemic LPS challenges resulted in increased brain IL-1β, TNF-α and IL-12 levels, despite attenuated systemic cytokine production. Each LPS challenge induced significant changes in burrowing behaviour. In contrast, brain IL-1β and IL-12 levels in *Salmonella typhimurium*-infected mice increased over three weeks, with high interferon-γ levels in the circulation. Behavioural changes were only observed during the acute phase of the infection. Microglia and cerebral vasculature display an activated phenotype, and focal intracerebral injection of LPS four weeks after infection results in an exaggerated local inflammatory response when compared to non-infected mice.

**Conclusions:**

These studies reveal that the innate immune cells in the brain do not become tolerant to systemic infection, but are primed instead. This may lead to prolonged and damaging cytokine production that may have a profound effect on the onset and/or progression of pre-existing neurodegenerative disease.

## Background

Humans and animals are regularly exposed to bacterial and viral pathogens that can have a considerable impact on our day-to-day living [1]. Upon infection, a set of immune, physiological, metabolic, and behavioural responses is initiated, representing a highly organized strategy of the organism to fight infection. Pro-inflammatory mediators generated in peripheral tissue communicate with the brain to modify behaviour [2], which aids our ability to fight and eliminate the pathogen. The communication pathways from the site of inflammation to the brain have been investigated in animal models and systemic challenge with lipopolysaccharide (LPS) or double-stranded RNA (poly I:C) have been widely used to mimic aspects of bacterial and viral infection respectively [3,4]. These studies have provided evidence that systemically generated inflammatory mediators signal to the brain via both neural and humoral routes, the latter signalling via the circumventricular organs or across the blood brain barrier (BBB). Signalling into the brain via these routes evokes a response in the perivascular macrophages (PVMs) and microglia, which in turn synthesise diverse inflammatory mediators including cytokines, prostaglandins and nitric oxide [2,5,6]. Immune-to-brain communication also occurs in humans who show changes in mood and cognition following systemic inflammation or infection, which are associated with changes in activity in particular regions of the central nervous system (CNS) [7-9]. While these changes are part of our normal homeostasis, it is increasingly evident that systemic inflammation has a detrimental effect in animals, and also humans, that suffer from chronic neurodegeneration [10,11]. We, and others, have shown that microglia become primed by ongoing neuropathology in the brain, which increases their response towards subsequent inflammatory stimuli, including systemic inflammation [12,13] Similar findings have been made in aged rodents [14,15], where it has been shown that there is an exaggerated behavioural and innate immune response in the brain to systemic bacterial and viral infections, but the molecular mechanisms underlying the microglial priming under these conditions is far from understood.

Humans and animals are rarely exposed to a single acute systemic inflammatory event: they rather encounter infectious pathogens that replicate *in vivo* or are exposed to low concentrations of LPS over a prolonged period of time. There is limited information on the impact of non-neurotrophic bacterial infections on the CNS and whether prolonged systemic inflammation will give rise to either a hyper-(priming) or hypo-(tolerance) innate immune response in the brain in response to a subsequent inflammatory stimulus.

In this study, we measured the levels of cytokines in the serum, spleen and brain as well as assessing sickness behaviour following a systemic bacterial infection using attenuated *Salmonella typhimurium* SL3261: we compared the effect to that of repeated LPS injections. We show that *Salmonella typhimurium* caused acute, transient behavioural changes and a robust peripheral immune response that peaks at day 7. Systemic inflammation resulted in a delayed increase in cytokine production in the brain and priming of microglia, which persisted up to four weeks post infection. These effects were not mimicked by repeated LPS challenges. It is well recognised that systemic bacterial and viral infections are significant contributors to morbidity in the elderly [16], and it has been suggested that primed microglia play a role in the increased clinical symptoms seen in patients with Alzheimer’s disease who have systemic inflammation or infections [11,17]. We show here that systemic infection leads to prolonged cytokine synthesis in the brain and also priming of brain innate immune cells to a subsequent focal inflammatory challenge in the brain parenchyma.

## Methods

### Mice

Adult female C57BL/6 or BALB/c mice (>8 weeks old) were obtained from Charles River, Margate, UK, and bred in house. Mice were housed in groups of five to ten, in plastic cages with sawdust bedding, for at least a week before testing. Food and water were available *ad libitum*. The holding room was temperature controlled (19 to 23°C) with a 12:12 h light–dark cycle (light on at 07:00 h). Females were used as they can be group-housed without the risk of outbreaks of aggression, and to conform to most of our previous work. BALB/c SCID mice were obtained from Harlan Laboratories Ltd (Harrow, UK) and bred in house. All procedures were performed under the authority of a UK Home Office License in accordance with the UK animals (Scientific Procedures) Act 1986, and after local ethical approval by the University of Southampton.

### Injection of LPS and infection with *Salmonella typhimurium*

In experiments investigating the effects of repeated LPS injections, mice received LPS derived from *Salmonella abortus equi* (L5886, Sigma-Aldrich, Poole, UK) at a dose of 500 μg/kg via intraperitoneal injection on three subsequent days at the same time of day (11:00 h). This dose of LPS was chosen for its reliable reduction in burrowing and reproducible cytokine response in the brain [12,18]. In experiments investigating the effects of a bacterial infection, mice were given a single intraperitoneal injection of 10^6^ colony-forming units (cfu) of attenuated *Salmonella typhimurium* strain SL3261 (generously provided by Dr H. Atkins, DSTL, Salisbury, UK).

### Burrowing and body weight

Burrowing was assessed as described previously [19]. Burrowing was measured between 1 and 3 hours after each LPS injection or at 1 to 3 h, 1d, 7d, and 21d after *Salmonella typhimurium* SL3261 infection. Body weight was measured immediately before the burrowing assay.

### Cytokine measurements

Blood samples (approximately 500 μl) were taken by cardiac puncture in terminally anaesthetized mice and collected in microfuge tubes. Samples were spun down and serum kept at −20 °C until further use. A three millimetre thick coronal section containing the dorsal hippocampus of the right hemisphere or spleen tissue was collected and homogenised in a TRIS buffer containing a protease inhibitor cocktail (150 mM NaCl, 25 mM Tris, 1% Triton X-100 pH 7.4, complete protease inhibitor cocktail (Roche Diagnostics GmbH, Mannheim, Germany)). Samples were centrifuged for 30 minutes at 13.000 rpm and supernatants assayed for total protein using a Pierce BCA protein assay kit (Thermo Fisher Scientific, Cambridge, UK). Cytokine levels in tissue and serum samples were assessed using MSD multiplex kit for mouse pro-inflammatory cytokines ((K15012B), Meso Scale Discovery, Gaithersburg, MD, USA), according to manufacturer’s instructions. Endotoxin levels in serum samples were assessed using Cambrex’ Limulus Amebocyte Lysate kit (Kinetic-QCL, Walkersville, MD, USA) according to manufacturer’s instructions.

### Priming of the brain innate immune response

Naïve mice or mice that were injected intraperitoneally with a single dose of 10^6^ cfu *Salmonella typhimurium* SL3261 were given a unilateral intracranial injection of LPS, four weeks after the systemic *Salmonella typhimurium* infection. The animals were anaesthetised with a mixture of ketamine and xylazine (Ketaset/Rompun 100/10 mg/kg body weight). LPS (100 pg in 1 μl) was injected into the dorsal hippocampus (from bregma: anterior-posterior − 2.0 mm, lateral + 1.7 mm, depth − 1.6 mm) using a glass micropipette (Sigma-Aldrich) as previously described [20]. At 24 h, mice were sacrificed, transcardially perfused with 0.9% heparinised saline and tissue collected for immunohistochemical analysis of microglial and endothelial cell activation.

### Immunohistochemistry

Immunohistochemistry was carried out on 10 μm-thick fresh frozen tissue sections. The tissue was air dried, fixed with cold ethanol, quenched with 1% hydrogen peroxide in phosphate-buffered saline (PBS), and blocked with 2% bovine serum albumin (BSA), 10% normal rabbit serum before overnight incubation with the primary antibodies: intercellular adhesion molecule-1(ICAM-1; YN1/1.7.4, Abcam, Cambridge, UK); vascular cell adhesion molecule-1 (VCAM-1; MVCAM A (429), Serotec, Oxford, UK); major histocompatibility complex class I (MHCI; ER-HR 52, Abcam); major histocompatibility complex class II (MHCII; M5/114.15.2, Abcam); CD11c (N418, a generous gift from Prof M. Glennie, Southampton, UK); CD11b (5 C6, Serotec); CD68 (FA11, Serotec), pan-Laminin (L9393, Sigma-Aldrich). Following incubation with the primary antibody, sections were washed and incubated with the appropriate biotinylated secondary antibody (Vector Labs, Peterborough, UK). Labelling was visualised using the avidin-biotin-peroxidase complex (ABC, Vector Labs), using 0.015% v/v hydrogen peroxide as substrate and diaminobenzidine (DAB) as chromogen (Sigma-Aldrich). For immunofluorescence, sections were incubated with 4% BSA, before overnight incubation with unconjugated primary antibodies and labelling was visualised by Alexa Fluor 488 or Alexa Fluor 546 conjugated secondary antibodies (Invitrogen, Paisley, UK). Mounted sections were cover-slipped using Prolong Gold antifade reagent with 4',6-diamidino-2-phenylindole (DAPI) (Invitrogen). Analysis of the immunohistological staining was performed using a Leica DM5000 microscope (Leica Micosystems, Milton Keynes, UK).

### Quantification of immunohistochemistry images

Images were captured at 20x optical zoom using QWin software by Leica Microsystems. All images were captured using identical exposure limit, gain, saturation, shading and filter. DAB staining within the images was identified using ImageJ with the Colour Deconvolution plugin to eliminate haematoxylin counterstain. Image threshold limit was lowered to a set level to remove background noise and kept constant for all images. Images were then converted to binary and the area of staining was measured: the fold increase in stained area was calculated using the contralateral hemisphere of LPS-injected mice as the control.

### Statistical analysis

Cytokine production was analysed by one-way analysis of variance (ANOVA) followed, if significant, by Tukey’s multiple comparison test using Graphpad Prism software (GraphPad Software Inc., La Jolla, CA, USA). Burrowing behaviour and body weight measurements were analysed by repeated ANOVA measures followed, if significant, by Tukey’s multiple comparison test using Graphpad Prism software (GraphPad). Immunohistochemistry results were analysed by two-way ANOVA followed by Bonferroni post-test using Graphpad Prism software (GraphPad). Values were expressed as means ± SEM. A *P* value < 0.05 was considered to indicate significant difference. Full ANOVA statistics are combined in Table S1 in Additional file 1.

## Results

### The effect of repeated LPS challenges on peripheral and central cytokines

**Figure 1:**
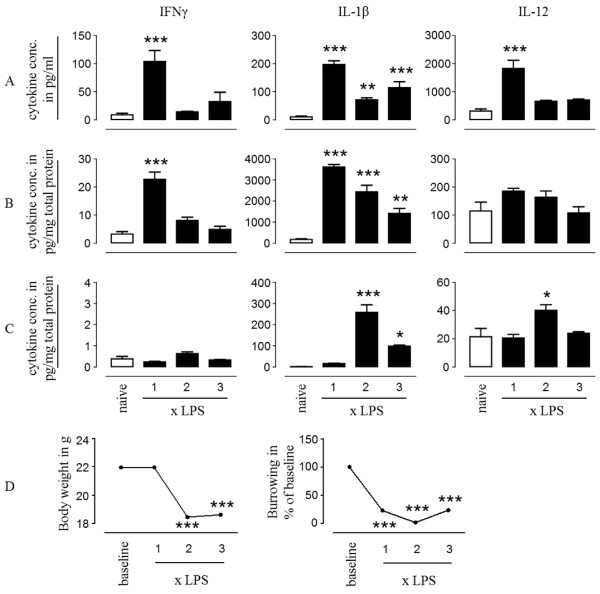
**Immune adaptation in response to multiple LPS challenges.** (**A**-**C**) Cytokine production in response to repeated systemic challenges with LPS. Levels of protein expression of inflammatory cytokines in serum (**A**), spleen (**B**), and brain (**C**) in saline or LPS- injected mice. LPS challenges were given on three consecutive days, with 24 h between each injection. Cytokine levels were measured 3 h after each LPS injection and compared to cytokine levels in naïve mice. (**D**) Changes in burrowing behaviour and body weight upon multiple LPS challenges. Error bars represent SEM from five animals per time point (*P* < 0.05 (*), *P* < 0.01 (**), *P* < 0.001 (***) versus naïve control). This experiment was repeated three times with similar results.

To investigate immune-to-brain communication following multiple systemic LPS challenges, we injected LPS intraperitoneally on three consecutive days and after each LPS challenge we measured burrowing activity and cytokines in the serum, spleen and brain. The first LPS challenge led to an increase in protein levels of interferon-gamma (IFN-γ), interleukin-1 beta (IL-1β) and IL-12 in the serum (Figure 1A, IFN-γ 7.1-fold *P* < 0.001, IL-1β 20-fold *P* < 0.001, IL-12 1.6-fold not significant (n.s.)) and the spleen (Figure 1B, IFN-γ 11.5-fold *P* < 0.001, IL-1β 18-fold *P* < 0.001, IL-12 5.8-fold *P* < 0.001). Similar changes were observed for IL-6, tumor necrosis factor-alpha (TNF-α), and keratinocyte*-*derived chemokine (KC) (data not shown). Upon the second and third challenge, serum and spleen levels of IFN-γ, TNF-α and IL-12 no longer increased in response to LPS, demonstrating induction of endotoxin tolerance (Figure 1A-B). In contrast to the periphery, protein levels of IFN-γ, IL-1β, IL-12 (Figure 1C) in the brain did not significantly increase relative to saline controls after the first systemic challenge with LPS, while a second challenge significantly increased the protein levels of IL-1β and IL-12 (Figure 1C, IL-1β 235-fold *P* < 0.001, IL-12 1.9-fold *P* < 0.05) . The third dose of LPS led to a reduced synthesis of IL-1β and IL-12 in the brain (Figure 1C, IL-1β 2.6-fold reduction *P* < 0.001, IL-12 1.7-fold reduction n.s.), similar to that observed in the periphery after multiple LPS challenges. Brain IFN-γ levels remain below 1 pg/mg protein, despite increased levels (up to 104 pg/ml) in the serum.

Increased systemic cytokine levels following LPS challenge has been associated with transient changes in sickness behaviour and body weight [18,21]. We observed that, despite a reduction in systemic cytokine levels, burrowing behaviour is reduced to below 30% (*P* < 0.001) of baseline burrowing upon each peripheral challenge with LPS (Figure 1D). The first LPS challenge induces a robust change in body weight measured 24 h after the injection (Figure 1D*P* < 0.001). A second or third challenge does not lead to a further significant decrease in body weight (Figure 1D).

### The effect of a bacterial infection on peripheral and central cytokines

**Figure 2:**
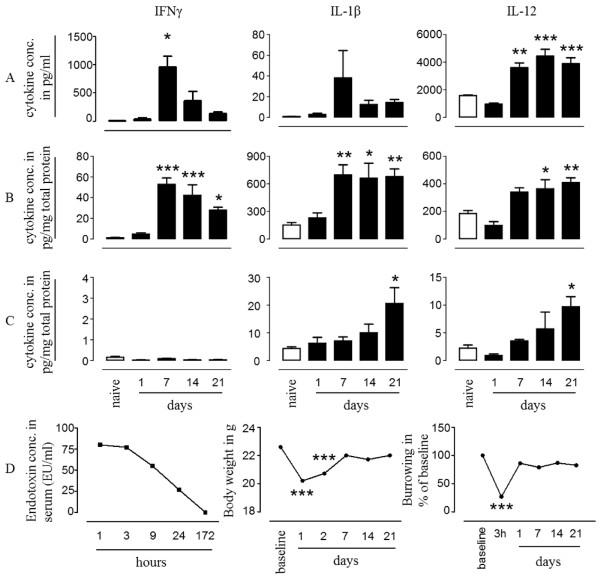
**Immune adaptation in response to SL3261 infection. **(**A**-**C**) Cytokine production in response to systemic challenge with *Salmonella typhimurium* SL3261. Levels of protein expression of inflammatory cytokines in serum (**A**), spleen (**B**), and brain (**C**) in naïve non-infected or SL3261-infected mice. Cytokine levels were measured at 1, 7, 14, and 21 days after the infection with 10^6^ cfu SL3261 and compared to cytokine levels in naïve mice. **(D)** Changes in burrowing behaviour, body weight and serum endotoxin levels upon SL3261 infection. Behaviour experiments were carried out with n = 5 per time point. Error bars represent SEM from five animals per time point (*P* < 0.05 (*), *P* < 0.01 (**), *P* < 0.001 (***) versus naïve control). This experiment was repeated two times with similar results.

LPS binds to and triggers TLR4 signalling in innate immune cells, and only mimics certain aspects of a bacterial infection. Therefore, we next measured cytokines levels in the periphery and brain following a non-neurotropic bacterial infection and assessed whether the cytokine levels correlate with behavioural and metabolic changes. Mice were inoculated with a sublethal dose of *S. typhimurium* strain SL3261 and cytokines levels in serum, spleen and brain were measured at 1, 7, 14 and 21 days. Burrowing, appearance and body weight was assessed on the day of infection and daily thereafter. At 24 h after SL3261 injection, we could not detect increased levels of pro-inflammatory cytokines in the periphery, while at day 7 protein levels of IFN-γ, IL-1β and IL-12 were increased in both serum (Figure 2A, IFN-γ 532-fold *P* < 0.001, IL-1β 55-fold n.s., IL-12 2.3-fold *P* < 0.01) and spleen (Figure 2B, IFN-γ 44-fold *P* < 0.001, IL-1β 4.3-fold *P* < 0.01, IL-12 1.8-fold n.s.) tissue. Serum levels of IFN-γ (959 ± 193 pg/ml) and IL-1β (38.2 ± 26.4 pg/ml) peaked at day 7, and circulating levels of IL-12 remained significantly elevated up to 21 days (3.6 ± 0.3 ng/ml, 4.4 ± 0.5 ng/ml, 3.9 ± 0.4 ng/ml at days 7, 14, and 21, respectively). In spleen tissue, we detected increased levels of all three cytokines from day 7 up to day 21 (Figure 2B). In the brain, the protein levels of the pro-inflammatory cytokines IL-1β, IL-12 (Figure 2C) and IL-6 (data not shown), were comparable to control levels when measured at day 7 post-infection, but levels of these cytokines increased gradually to become significantly increased at 21 days post-infection (Figure 2C, IL-1β 4.7-fold *P* < 0.05, IL-12 4.4-fold *P* < 0.05). Brain levels of IFN-γ remained below 1 pg/mg protein during the entire three-week period post-infection (Figure 2C).

Burrowing was significantly reduced to 30% (*P* < 0.001) of baseline when measured 1 to 3 h after administration of SL3261, and after 24 h, these changes reverted back to baseline levels (Figure 2D). Mice showed transient changes in appearance during 1 to 3 h (for example, hunched posture, piloerection), which were no longer observed 24 h after infection. The changes in burrowing and appearance coincided with endotoxin levels in the serum of the animals (Figure 2D). Inoculation of mice with SL3261 resulted in a robust loss of body weight (Figure 2D, *P* < 0.001) and this was restored to baseline levels at day 7 post-infection (Figure 2D). At this stage, the lymphoid organs (spleen and draining lymph nodes) had markedly increased their size to approximately five times their original size (data not shown). These data show that a bacterial infection induces a strong immune activation in the periphery, and a delayed but long-lasting cytokine response in the brain. The increased levels of cytokines in the periphery and brain did not correlate with anhedonia behaviour assessed by burrowing (Figure 2).

### Cellular changes in the brain

To further characterise the impact of a systemic bacterial infection on the brain, we examined coronal sections between 1.8 and 2 mm posterior to bregma for a range of molecules typically expressed on activated endothelium (ICAM-1, VCAM-1, MHCI, MHCII) or microglia/macrophages (CD68, CD11b, MHCI, MHCII) at 1, 7, 14 and 21 days following infection with SL3261.

### Changes in cerebral vasculature

**Figure 3:**
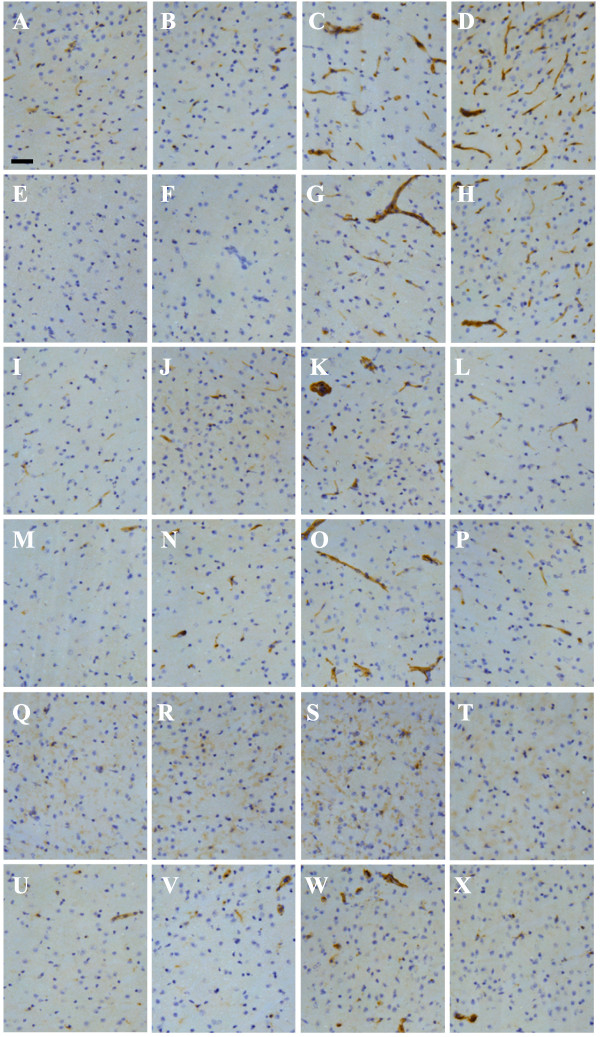
**Kinetics of immune marker expression after SL3261 infection.** Immune markers of vascular endothelial cells and microglia/macrophages (MHCI (**A**-**D**), MHCII (**E**-**H**), ICAM-1 (**I**-**L**), VCAM-1 (**M**-**P**), CD11b (**Q**-**T**), and CD68 (**U**-**X**)) at 1d (**B**, **F**, **J**, **N**, **R**, **V**), 7d (**C**, **G**, **K**, **O**, **S**, **W**), and 21d (**D**, **H**, **L**, **P**, **T**, **X**) after a systemic challenge with *Salmonella typhimurium* SL3261 compared to naïve non-infected mice (**A**, **E**, **I**, **M**, **Q**, **U**). Representative images from the dorsal lateral geniculate nucleus of the thalamus are shown. Phenotype analysis was carried out with n = 3 mice per time point. Scale bar = 50 μm.

**Figure 4:**
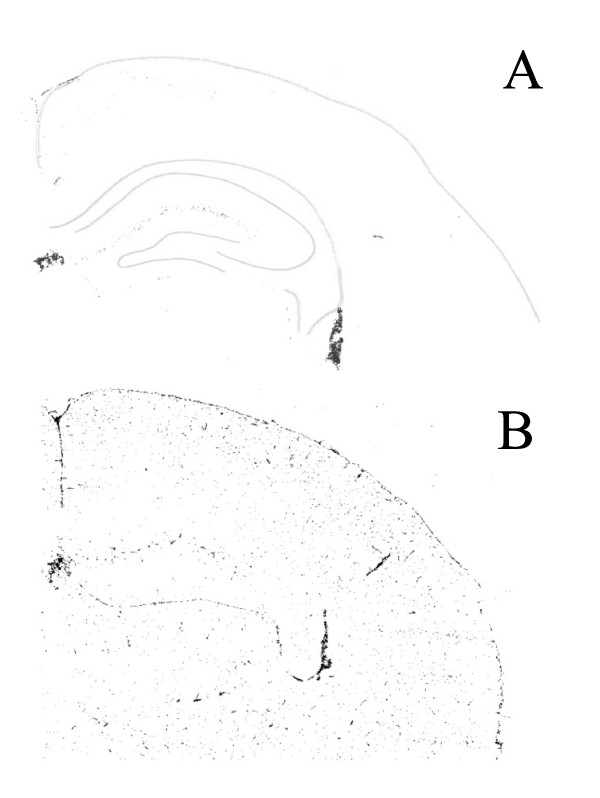
**MHCII expression in the brain after systemic SL3261 infection.** Brains from naïve mice (**A**) and mice that were infected with SL3261, 7 days previously (**B**) were cut through the dorsal hippocampus and thalamus and analysed for MHCII expression. The image of the naïve brain was overlaid with an outline of the brain and the cell fields of the dorsal hippocampus to aid the orientation within the image.

The most profound change on the cerebral vasculature was the up-regulation of MHCI and MHCII. Low levels of MHCI can be detected on the cerebral vasculature of naïve control mice (Figure 3A), while MHCII is undetectable (Figure 3E). At 24 h post-infection, no changes in expression levels of these molecules were observed (Figure 3B and F). However, from day 7 after infection the cerebral vasculature, including veins, arteries and capillaries, as judged by their morphology and size, stained positive for both MHCI and MHCII (Figure 3C and G). Increased expression of MHCII molecules was conspicuous in the cortex and hippocampus (Figure 4), and levels remained readily detectable on the cerebral vasculature throughout the three-week time course. The administration of a single LPS challenge did not induce MHCII on the brain vasculature, measured at day 3 and 7, nor did multiple LPS challenges, measured 3 h after the final injection (data not shown). The cell adhesion molecules VCAM-1 and ICAM-1 were both expressed at low levels on the brain vasculature of naive mice (Figure 3I and M). Increased expression of VCAM-1 and ICAM-1 was observed from 24 h post-infection (Figure 3J and N), but the most prominent changes were observed at day 7 after infection (Figure 3K and O). At three weeks, the expression levels of the adhesion molecules had decreased to similar levels as observed at day 1 (Figure 3L and P).

### Changes in parenchymal cells

Analysis of microglia/macrophage markers suggested that microglia also respond to a systemic challenge of SL3261. At day 7 after infection, we observed a modest increase in CD11b expression in mice inoculated with SL3261 (Figure 3S), when compared to naïve control mice (Figure 3Q). Infection of mice with SL3261 also led to increased expression of CD68 in perivascular macrophages and microglia (Figure 3W), while in naïve mice, CD68 is conspicuously expressed in perivascular macrophages (Figure 3U). The changes in CD11b and CD68 expression returned to baseline levels at three weeks (Figure 3T and X). The morphology of the microglia remained ramified with fine processes, despite changes in CD11b and CD68 expression.

### Cellular changes in the brain of SCID mice

**Figure 5:**
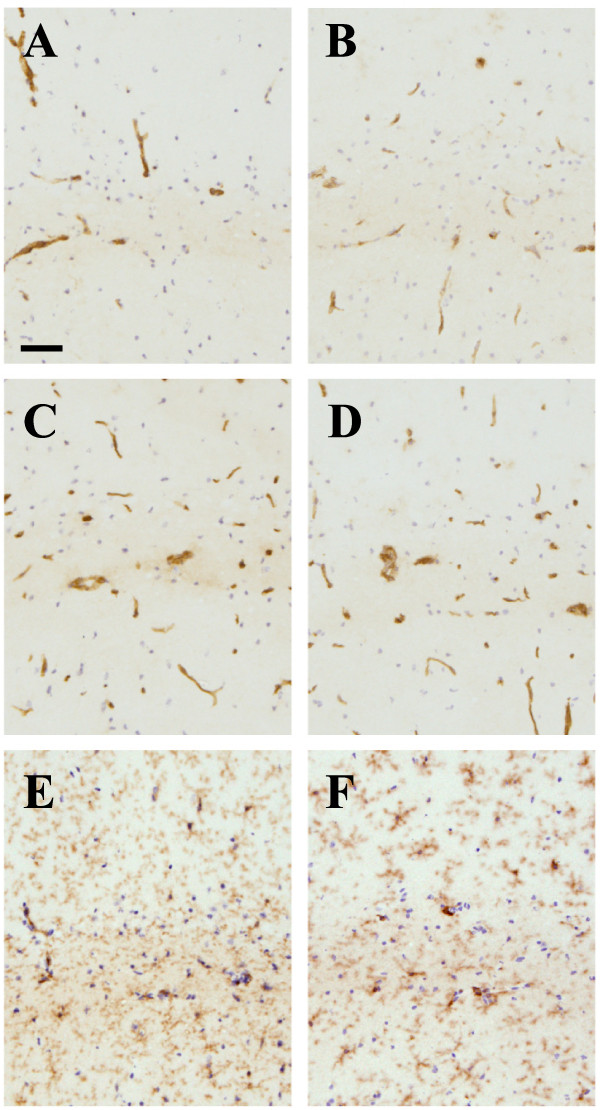
**Immune marker expression in SCID mice after systemic infection with SL3261.** Immune markers of the vascular endothelial cells and microglia/macrophages (MHCII (**A**-**B**), MHCI (**C**-**D**), and CD11b (**E**-**F**)) in BALB/c (**A**, **C**, **E**) or BALB/c SCID (**B**, **D**, **F**) mice measured at day 5 after a systemic infection with SL3261. Due to more severe clinical symptoms in the SCID mice following SL3261, mice were sacrificed at day 5. BALB/c wild-type mice were analysed at the same time point for comparison. The images are representative examples of immune marker activation of n = 5 animals per group. Scale bar = 50 μm.

To determine whether the phenotypic changes of cerebral vascular cells and microglia are dependent on innate immune responses, we compared the cellular changes in the brain of BALB/c SCID and BALB/c control mice, following systemic SL3261 infection. As seen in C57BL/6 J mice, we observed increased expression of MHCII (Figure 5A and B) and MHCI (Figure 5C and D) on cerebral vascular cells in both BALB/c mouse strains, analysed 5 days post-infection. In addition, no apparent differences were found in CD11b immunoreactivity on microglia in the brain parenchyma between SCID or control mice infected with SL3621 (Figure 5E and F).

### Priming of the brain innate immune response

**Figure 6:**
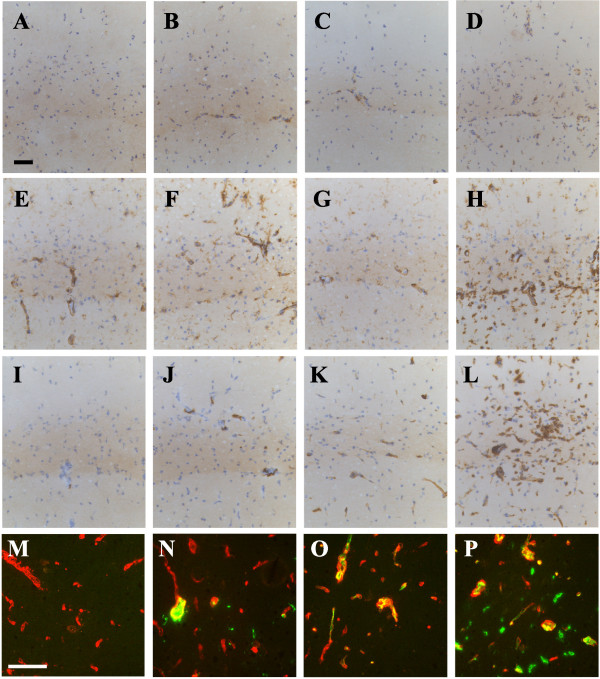
**Primed innate immune response in the brain following a systemic bacterial infection.** Phenotypic changes after intracerebral injection of 100 pg of LPS in naïve mice (**A**, **B**, **E**, **F**, **I**, **J**, **M**, **N**) or mice pretreated with SL3261 (**C**, **D**, **G**, **H**, **K**, **L**, **O**, **P**). Representative images of immune marker expression (CD11c (**A**-**D**), CD68 (**E**-**H**), MHCII (**I**-**L**) in the injected hemisphere (**B**, **D**, **F**, **H**, **J**, **L**, **N**, **P**) or contralateral site are shown. Panels M-P show a double immunofluorescent stain for MHCII (green) and laminin (red). n = 5 animals per group, black scale bar = 50 μm, white scale bar = 75 μm.

**Figure 7:**
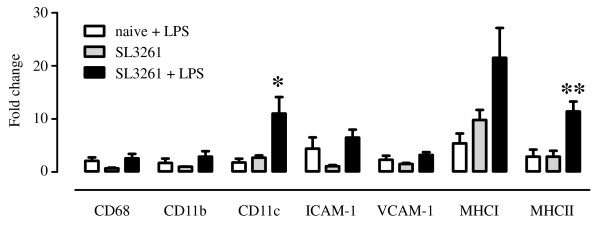
**Immune marker expression after intracerebral injection of LPS.** LPS was injected intracerebrally into one hemisphere of naïve mice or mice that had been infected with SL3261 four weeks previously. The figure shows the fold increase and SEM of immune marker expression over the naïve uninjected section. Data was analysed by two-way ANOVA and significant interaction is shown (*P* < 0.05 (*), *P* < 0.01 (**), n = 4-5).

The immunohistochemical analysis suggests that microglia/macrophages become transiently activated after infection with SL3261, and appear to return to their original morphology and phenotype by three weeks. However, brain cytokines remain elevated, suggesting an altered innate immune response in the brain. To investigate whether the innate immune cells in the brain are ‘primed’ by systemic infection, we challenged mice with a focal intrahippocampal, unilateral injection of 100 pg of LPS four weeks after the initial systemic infection. In naïve animals, LPS injection led to a small, but detectable up-regulation of the expression of a number of myeloid markers on microglia and macrophages (Figure 6A, E, and I) compared to the uninjected site (Figure 6B, F, and J). A few microglia displayed thicker processes and hypertrophic cell bodies (Figure 6F). Quantification of the data show that microglia in the LPS-injected hemisphere of naïve mice exhibit a 2-fold increase in CD68 expression (F_(1,10)_ = 7.09 *P* = 0.0238) relative to uninjected brain (Figure 7) and SL3261-treated animals showed a 2.6-fold increase of CD68 (F_(1,10)_ = 7.09 *P* < 0.05) following an intracerebral injection of LPS and displayed an activated morphology with retracted processes, but these changes were not different from similarly treated naïve mice (F_(1,10)_ = 0.53 n.s.). Similar results were observed for CD11b expression levels (Figure 7). In contrast, focal injection of LPS into the brain of previously SL3261-infected animals led to higher expression levels of CD11c (Figure 6A-D and 7, interaction: F_(1,12)_ = 5.59 *P* < 0.05), MHCII (Figure 6L and 7, interaction: F_(1,14)_ = 10.0 *P* < 0.01), and MHCI (Figure 7, interaction: F_(1,14)_ = 1.11 n.s.). Microglia or macrophages in the parenchyma showed expression of CD11c, which was not detected on microglia of naïve mice (Figure 6A-D). In addition, there appeared to be an increased number of CD11c + cells in the perivascular space of SL3261 pre-treated mice, suggesting possible recruitment of myeloid cells from the circulation (Figure 6D). The cerebral vasculature of mice infected with SL3261 four weeks previous to the LPS challenge remained positive for MHCII (Figure 6K and O) and intracerebral injection of LPS induced MHCII expression on parenchymal microglia/macrophages. This observation was confirmed by a double staining with laminin to differentiate MHCII ^+^ microglial cells from MHCII ^+^ endothelial cells of the vasculature (Figure 6P).

## Discussion

A systemic infection activates multiple physiological pathways that aid in the elimination of the pathogen and recovery of the host. These include innate and acquired immune responses as well as the activation of communication pathways between the immune system and the brain to co-ordinate essential metabolic and behavioural changes. The mechanisms underlying immune-to-brain communication following a single dose of LPS are well described [22], but information on real bacterial infections restricted to peripheral tissues is still sparse. This is surprising, given how frequently systemic infections occur in the population and their potential to exacerbate brain disease [11,23]. In the current study, we compared the cytokine response in the periphery and the brain of mice that received either multiple doses of *S. abortus equi*-derived LPS or a bacterial infection with attenuated *Salmonella typhimurium* (SL3261). A single LPS injection induced high systemic cytokine levels with associated behavioural changes, but no detectable levels of IL-1β and IL-12 protein in the brain. A second LPS challenge induced significant protein levels of pro-inflammatory cytokines in the brain, despite reduced peripheral cytokine expression. A subsequent third challenge with LPS resulted in a reduced synthesis of cytokines both systemically and in the brain, indicative of tolerance in both compartments. The levels of cytokines did not correlate in a simple way, with a deficit in burrowing behaviour, a measure of anhedonia, which was reduced after each LPS challenge, suggesting that the burrowing is not controlled by cytokines alone and not altered during endotoxin tolerance.

When we assessed the effect of a bacterial infection*,* we observed acute behavioural changes that rapidly resolved. Despite these transient changes in behaviour, there was clear evidence of sustained immune activation in both the periphery and the brain. Cytokines in the serum, including IFN-γ and IL-1β were significantly increased at day 7, and raised levels of IL-1β and IL-12 in the spleen were present up to three weeks post-infection. In addition to cytokine production, we observed up-regulation of ICAM-1, VCAM-1, MHCI, and MHCII on the cerebral vasculature and increased levels of CD11b and CD68 on microglia. These observations demonstrate that repeated systemic LPS injections and a bacterial infection both result in immune activation in the brain but with different kinetics, different magnitude and differential cell involvement in the brain including endothelium, perivascular macrophages and microglia.

### Priming versus tolerance

Mounting an immune response to infection is essential for survival, but excessive or prolonged inflammatory responses can result in bystander tissue damage and septic shock. One mechanism to regulate innate immune responses is endotoxin tolerance, which is characterised by a transient state of cellular, metabolic and behavioural hypo-responsiveness to subsequent or chronic systemic infections [24]. Stimulation of TLR4 results in signalling of nuclear factor kappa B (NFκB), which induces gene transcription of pro-inflammatory cytokines including TNF-α, IL-12 and IL-1β, while a second stimulation with LPS results in a repression of these genes leading to reduced lethality, fever, and anorexia [25-27]. It has been shown that LPS-induced cytokine expression still occurs in the brain during endotoxin tolerance, when cytokines in the periphery are no longer induced [28,29] and our results are in agreement with these observations. Faggioni *et al*. suggested that tolerance induction in the brain requires direct stimulation of TLR4-expressing cells. This is supported by experiments where LPS was directly injected into the brain, showing reduced TNF-α production upon restimulation [29]. Other studies have shown that systemic exposure to LPS induced endotoxin tolerance to a different bacterial strain [30]. These changes were independent of peripheral cytokine levels and long lasting, as tolerance was still observed 3 weeks after the first challenge. However, it should be noted that the high dose of LPS (1 mg/kg) may have altered BBB permeability allowing exposure of perivascular macrophages or microglia to LPS. Although microglia are a likely source of increased pro-inflammatory cytokines following a systemic infection, we cannot rule out other cell types. Endothelial cells express functionally significant amounts of TLR4 and are capable of producing inflammatory cytokines and chemokines [31]. *In vitro* studies show that cytokines are continuously produced by endothelial cells after repeated LPS treatment [32], which implies that endothelial cells do not become tolerant to LPS. Recent studies have suggested that epigenetic changes play a role in endotoxin tolerance [33,34], and that gene selective programming and histone modification determines the threshold for subsequent cell activation [33-35]. In contrast to most macrophages in the body, microglia are kept in a quiescent state and it is possible that exposure to LPS induces chromatin remodelling, which then allows further activation by a secondary challenge. Similar observations have been made by Stalder et al. [36], who demonstrated that microglia were the major producers of IL-12 in the brain after a second systemic LPS challenge in contrast to spleen where high levels of IL-12 were produced after the first challenge. The degree of tolerance may involve numerous variables including the strain of LPS used in the first and subsequent challenges [30] and the delay between the challenges.

Microglia and macrophages in the brain parenchyma of SL3261-pretreated mice showed increased levels of MHCII and CD11c, after intracerebral LPS challenge, which was not seen in similarly challenged naïve mice. It has been reported that microglia express CD11c under inflammatory conditions, therefore CD11c positive cells in SL3261-pretreated and LPS-restimulated brain can be both activated microglia and/or recruited macrophages from the circulation [37,38]. Previous studies investigating the impact of the environment on the immune response of the brain parenchyma to a viral vector and reporter gene synthesis showed a similar hyper-responsive effect. Animals exposed to a conventional animal house environment gave a more robust inflammatory response to the viral vector and reduced reporter gene synthesis when compared to animals raised and living in a specific-pathogen-free environment [39]. The fact that the innate immune response appears to be influenced by prior exposure many days or even weeks previously is suggestive of an innate immune memory. Netea *et al*. [40] have reviewed this phenomenon in plants, invertebrates and vertebrates and suggest the existence of a trained immunity, mediated by epigenetic changes of the immune system. Furthermore, recent observations show that early-life infections can alter the threshold of IL-1β production in the hippocampus via long-lasting priming of microglia [41]. Based on these observations we hypothesise that epigenetic changes may control the innate priming observed in our model.

### The impact of *S. typhimurium* on the CNS, innate versus adaptive immunity

The immune response to systemic infection with wild-type *S. typhimurium* and its attenuated aroA mutant strain SL3261 is well documented [42,43]. Macrophages and neutrophils play a key role in the initial response to infection by secreting the cytokines IL-12, IL-18, and TNF-α. IL-12 contributes to the establishment of a protective T helper 1 (Th1) immune response and mediates host resistance by inducing IFN-γ synthesis in natural killer (NK)-cells and macrophages [44-46]. Clearance of the bacteria requires MHC-II restricted, SL3261-specific CD4^+^ T cells that become detectable at 7 days post-infection [42]. These immune responses are not observed after multiple LPS challenges, and a major finding of our study is to highlight that repeated LPS challenges poorly mimic the peripheral immune response to a bacterial infection. Our study shows increased levels of IL-12 up to three weeks post-infection and increased levels of IFN-γ in serum and spleen that peak at day 7, while repeated LPS induced a blunted cytokine response in the periphery and did not result in increased expression of MHCI and MHCII on cerebral endothelial cells. We detected acute changes in burrowing that normalized after 24 h. The behavioural changes coincided with detectable circulating levels of LPS in the serum, making it possible that systemic LPS induced the behavioural changes directly by engaging TLR4 on cerebral endothelial cells.

### Long-term consequences of systemic inflammation

A striking effect following systemic infection with SL3261 was the prolonged up-regulation of MHCI and MHCII expression on the cerebral vasculature. To our knowledge, this is the first study to report these long-lasting alterations to cerebral vasculature in response to non-neurotropic bacterial infection. IFN-γ is a major regulator of MHC molecules on cerebral endothelium and it has been shown that IFN-γ up-regulates MHCII on cerebral endothelium in culture [47] and following infection with *Toxoplasma gondii* (46). Our data using SCID mice suggest that innate immune cells, such as macrophages or NK cells, are the likely cellular source of this cytokine. The functional consequences of increased MHCI and MHCII expression on cerebral endothelium following SL3261 are unknown. One possibility is that increased expression of MHC molecules contributes to a protective mechanism to regulate immune activation in the CNS, by inducing T cell tolerance in the absence of co-stimulatory molecules such as CD40 or CD80 (data not shown). A similar lack of co-stimulatory molecules has been shown *in vitro* following stimulation of primary human endothelial cells [48]. It has also been shown that MHCI expression on endothelium can act as a molecular address for CD8 cell invasion into the brain parenchyma without activation of the cells [49].

Systemic infection with *Mycobacterium tuberculosis* (Bacillus Calmette-Guérin, BCG) leads to acute changes in locomotor activity, followed by depressive symptoms at 7 days that coincide with an increase in indoleamine 2,3-dioxygenase (IDO) and increased mRNA levels of IFN-γ, IL-1β and TNF-α in the brain [50,51]. BCG and *S. typhimurium* infect their host cells in a similar way and it is likely that they share similar immune-to-brain communication pathways.

## Conclusions

Our study shows that systemic infection with non-neurotropic *S. typhimurium* has long-term consequences for homeostasis in the CNS, due to changes in the cerebral vasculature and priming of the innate immune response. It is well recognised that systemic bacterial and viral infections are significant contributors to morbidity in the elderly. A number of studies have described morphologically activated microglia in the aged rodent and human brain with increased levels of MHCII and CD68 expression [52,53]. Altered expression of immunoregulatory molecules, such as CD200 or fractalkine on neurons is one explanation for these microglial phenotype changes. As an alternative or additional factor, we propose that the morphologically activated microglia of the aged brain may be primed by low-grade systemic inflammation experienced throughout life.

## Competing interests

The authors declare that they have no competing interests.

## Authors’ contribution

UP, SB and JT carried out the experiments and performed statistical analysis. JT and VHP participated in the design and coordination of the study. JT, UP and VHP wrote the manuscript. All authors read and approved the final manuscript.

## Supplementary Material

Additional file 1 **Table S1.** Showing details of statistical analysis. The table gives an overview on statistics of all ANOVA test runs that have been performed to analyse the data presented in this manuscript. (PDF 36 kb) Click here for file
