# Supplementary material for: Long-term impact of systemic bacterial infection on the cerebral vasculature and microglia
Source: J Neuroinflammation. 2012 Jun 27;9:146. doi: 10.1186/1742-2094-9-146 (PMC3439352; doi:10.1186/1742-2094-9-146)
Supplement: Additional file 1 — Table S1. Showing details of statistical analysis. The table gives an overview on statistics of all ANOVA test runs that have been performed to analyse the data presented in this manuscript. (PDF 36 kb) [file 1742-2094-9-146-S1.pdf]

1-way ANOVA LPS challenge

	serum	spleen	brain
IFN-γ	$F_{(2,15)}$ = 13.10 p = 0.0002	$F_{(2,15)}$ = 30.27 p <0.0001	$F_{(2,15)}$ = 5.056 p = 0.0129
IL-1β	$F_{(2,15)}$ = 48 p <0.0001	$F_{(2,15)}$ = 56.45 p <0.0001	$F_{(2,15)}$ = 40.97 p <0.0001
IL-12	$F_{(2,15)}$ = 17.04 p <0.0001	$F_{(2,15)}$ = 2.643 n.s.	$F_{(2,15)}$ = 5.409 p = 0.101

1-way ANOVA Salmonella infection

	serum	spleen	brain
IFN-γ	$F_{(3,20)}$ = 11.82 p < 0.0001	$F_{(3,20)}$ = 16.95 p <0.0001	$F_{(3,20)}$ = 5.820 p = 0.0028
IL-1β	$F_{(3,20)}$ = 1.533 n.s.	$F_{(3,20)}$ = 7.443 p = 0.0008	$F_{(3,20)}$ = 4.312 p = 0.0112
IL-12	$F_{(3,20)}$ = 21.53 p <0.0001	$F_{(3,20)}$ = 11.15 p <0.0001	$F_{(3,20)}$ = 4.604 p = 0.0085

repeated measures ANOVA for weight loss and burrowing behaviour

	body weight	burrowing
LPS	$F_{(1,16)}$ = 26.68 p < 0.0001	$F_{(2,24)}$ = 288.6 p <0.0001
Salmonella	$F_{(4,20)}$ = 11.96 p <0.0001	$F_{(4,20)}$ = 11.64 p <0.0001

2-way ANOVA IHC quantification

	Salmonella infection	LPS injection	Interaction
CD68	$F_{(1,10)}$ = 0.04 p = 0.8443	$F_{(1,10)}$ = 7.09 p = 0.0238	$F_{(1,10)}$ = 0.53 p = 0.4826
CD11b	$F_{(1,10)}$ = 0.63 p = 0.4441	$F_{(1,10)}$ = 2.96 p = 0.1161	$F_{(1,10)}$ = 0.62 p = 0.4509
ICAM-1	$F_{(1,14)}$ = 0.69 p = 0.4212	$F_{(1,14)}$ = 11.78 p = 0.0040	$F_{(1,14)}$ = 0.61 p = 0.4478
VCAM-1	$F_{(1,14)}$ = 2.21 p = 0.1596	$F_{(1,14)}$ = 9.24 p = 0.0088	$F_{(1,14)}$ = 0.14 p = 0.7106
MHCI	$F_{(1,14)}$ = 12.98 p = 0.003	$F_{(1,14)}$ = 5.42 p = 0.0354	$F_{(1,14)}$ = 1.11 p = 0.3104
MHCII	$F_{(1,14)}$ = 24.81 p = 0.0002	$F_{(1,14)}$ = 24.81 p = 0.0002	$F_{(1,14)}$ = 10.0 p =0.0069
CD11c	$F_{(1,12)}$ = 11.68 p = 0.0051	$F_{(1,12)}$ = 8.19 p = 0.0143	$F_{(1,12)}$ = 5.59 p = 0.0358

Abbreviations: ANOVA, analysis of variance; ICAM-1, intercellular adhesion molecule-1; IFN, interferon; IHC, immunohistochemistry; IL, interleukin; LPS, lipopolysaccharide; MCHI/I, major histocompatibility Class I/I; n.s., not significant; VCAM-1, vascular cell adhesion molecule-1.
